# Mycobacterial heparin-binding haemagglutinin adhesion-induced interferon & antibody for detection of tuberculosis

**Published:** 2011-04

**Authors:** Zhaogang Sun, Lihui Nie, Xuxia Zhang, Yan Li, Chuanyou Li

**Affiliations:** *Department of Bacteriology & Immunology, Beijing Tuberculosis & Thoracic Tumor Research Institute, Beijing, PR China*

**Keywords:** Binding haemagglutinin, differential diagnosis, heparin, interferon-γ level, serodiagnostic test, tuberculosis

## Abstract

**Background & objectives:**

Mycobacterial heparin-binding haemagglutinin adhesin (HBHA) plays an important role in humoral and cellular immune response and is a potential diagnostic tool for tuberculosis (TB) serodiagnosis. This study was carried out to assess the usefulness of HBHA in TB clinics for differential diagnosis of pulmonary and extra-pulmonary TB (PTB, EPTB).

**Methods:**

In this study, 165 outpatients and 133 healthy volunteers were included to investigate the role of HBHA in TB diagnosis including the serodiagnostic tests and the interferon-γ release assays (IGRAs). The healthy volunteers were all without BCG vaccination including 73 subjects with purified protein derivative (PPD) (-) and 60 ones with PPD (+) (that is P-B- and P+B-). Of all the 165 outpatients 77 were PTB and 88 were EPTB. HBHA protein was used for serodiagnostic tests and IGRAs in peripheral blood mononuclear cells.

**Results:**

HBHA-specific antibody levels in the serum of healthy subjects were significantly different from the patients with PTB or EPTB (*P*<0.05). HBHA specific antibody levels in PTB patients could differentiate from EPTB with limited sensitivity (77.08%; 95%CI, 62.69 to 87.97%) and specificity (87.50%; 95%CI, 74.75 to 95.27%). IFN-γ levels in the healthy (P+B- and P-B-) groups were significantly different (*P*<0.01) with a detection sensitivity of 84.8% (95%CI, 68.54 to 93.02%) and specificity of 80.7% (95%CI, 65.22 to 92.62%). The PTB and EPTB subjects showed no difference in IFN-γ production.

**Interpretation & conclusions:**

HBHA serodiagnostic test with IGRAs had the limited potential for use as auxiliary tools for the differential diagnosis of PTB and EPTB, since both methods showed low sensitivity and specificity.

Tuberculosis (TB) remains a major problem for most parts of the world, with nearly two million people being killed annually^1^. A better definition of the humoral and cellular immune responses to *Mycobacterium tuberculosis* antigens such as heparin-binding haemagglutinin adhesin (HBHA) may have an important impact on the differential diagnosis and vaccine development.

The mycobacterium HBHA is a surface-associated protein^2^. Native HBHA (nHBHA) is modified at the C-terminal domain with a 20-26 methyl group after translation^3^, while the recombinant HBHA (rHBHA) produced by *Escherichia coli* has no methyl groups^4^. In pathogenic mycobacteria, HBHA is an adhesin for nonphagocytic cells^2^,^3^,^5^ and is implicated in extra-pulmonary (EPTB) dissemination of *M. tuberculosis* from the lung, following initial host infection^3^,^6^. HBHA can induce high levels of anti-HBHA antibodies in most patients with active TB, but not in healthy subjects with latent infection, BCG-immunized subjects and healthy controls^7^. HBHA can also induce T-lymphocytes to produce large amounts of HBHA specific interferon gamma (IFN-γ) from healthy human individuals as compared to those with active TB^4^,^8^. Tests based on *in vitro* release of IFN-γ by memory T lymphocytes have been introduced for the diagnosis of *M. tuberculosis* infection using *M. tuberculosis*-specific antigens, such as 6 kilodaltons ESAT-6 and culture filtrate protein CFP-10 of 10 kilodaltons^9^,^10^. More recently, the HBHA-induced IFN-γ release assay (IGRA) has been shown a great potential for the diagnosis of latent TB infection^7^,^11^. Recent progress in protein purification systems has paved the path of purifying nHBHA from whole crude bacterium proteins^12^.

Although HBHA has been known to be associated with EPTB and it can stimulate IFN-γ production in individuals with latent TB infection, little is known about the role of HBHA in the differential diagnosis of EPTB and pulmonary (PTB) by the combination of humoral and cellular immune response. In this study we compared the different levels of HBHA specific antibodies produced by patients with PTB or EPTB, and healthy subjects without BCG vaccination with purified protein derivative (PPD) (+) or PPD (-) according to their tuberculin skin tests (TST). In addition, IFN-γ produced by peripheral blood mononuclear cells (PBMCs) from these groups was also compared.

## Material and Methods

*Ethical agreement and participants consent*: This study protocol was approved by the ethical committee of Beijing Tuberculosis & Thoracic Tumor Research Institute, Beijing, China. Participation in the investigation from Match 2005 to April 2008 was voluntary. Each participant’s written consent for the questionnaire and for the blood specimen collection (4 ml) was obtained.

*Bacterial strain and growth conditions*: M. bovis BCG (ATCC 35737) was obtained from the Mycobacterium Research Center of Japan, Tokyo. Growth conditions for M. bovis BCG (ATCC 35737) followed the described method^7^.

*Antigens*: By using the plasmid of pET32a (+) the HBHA gene was expressed in *E. coli* BL21(DE3) with a molecular weight of 43 K Dalton^2^,^8^. DNA manipulation and plasmid construction were as the following description. Restriction enzymes, T4 DNA ligase, and other molecular biology reagents were purchased from Takara, Japan and were used as recommended by the suppliers. All DNA manipulations were carried out as described by Sambrook and Russell^13^. Mycobacterial chromosomal DNA was prepared as described^14^, with the annealing temperature at 70 °C. PCR was performed in a Perkin-Elmer thermal cycler model 480 (USA), using 50 ng of Mycobacterial chrosomal DNA and 1 µg of each primer (sense primer: 5’ AAC GAA TTC ATG GCT GAA AAC TCG AAC ATT 3’; anti-sense primer: 5’ ACG GGA TCC CTA CTT CTG GGT GAC CTT CTT 3’). Based on the sequence (NC_000962) in GenBank (*http://www.ncbi.nlm.nih.gov/nuccore/NC_000962*), the primers were designed using Oligo 6.0 software (Molecular Biology Insights, USA). Cloning steps were performed in *E. coli* pUC18 selected with ampicillin (50 µg/ml) to obtain the plasmid pUC18-HBHA. Then HBHA DNA was sequenced (Takara Co., Japan).

Recombinant histidine-tagged rHBHA was purified from *E. coli* BL21 (DE3) (pET32a-HBHA) by nickel-sepharose resin column (Novagen, USA) as indicated previously^8^,^15^. The nHBHA was purified from BCG by heparin-sepharose (Amersham Biosciences, USA)^2^ and high-pressure liquid chromatography^9^. They were verified by SDS-PAGE and Western blot methods^16^.

*Antiserum*: Antiserum to rHBHA was collected from mice (18~20 g, Laboratory Animal Center of Chinese Academy of Medical Science) vaccinated four times at intervals of one week with approximately 200 µg of the rHBHA fusion protein emulsified in Freund’s incomplete adjuvant (Invitrogen Corp., USA).

*Subjects*: A total of 165 TB patients (outpatients) and 133 healthy volunteers (age ranging from 20 to 55 yr) were selected randomly, in this study to investigate the role of HBHA in TB diagnosis. The healthy volunteers were without BCG vaccination [73 with purified protein derivative (PPD) (-) and 60 PPD+ *i.e*, is P-B- and P+B-]. The healthy volunteers were selected randomly were students from Tsinghua University, Beihang University and the policemen from the General Team of Beijing Armed Police Force after TST test and further X-ray check was done for PPD+ subjects. TST was determined with diameter of induration size > 10 mm after an intradermal injection of 5 tuberculin units of PPD (Xiangrui Corp., Beijing, China) and the results of the TST were read after 48 h^10^ (Table I) provides the details of the sex, age and the number of subjects in different groups (PTB, EPTB, P+B- and P-B-). EPTB cases included tuberculosis lymphadenitis (7 cases), pleural tuberculosis (9 cases), skeletal tuberculosis (14 cases), abdominal tuberculosis (16 cases), endometrial tuberculosis (5 cases), female pelvic peritoneal tuberculosis (3 cases), tuberculous pericarditis (8 cases) tuberculosis of intestines (3 cases), cutaneous tuberculosis (3 cases) and tuberculosis meningitis (7 cases). Serum samples were transferred to the laboratory at -20 °C and stored at -80 °C.

**Table I.** Baseline characteristics of out-patients (n=165) and healthy (n=133)

Characteristic	Serodiagnostic tests				Interferon-γ release assays			
	Healthy subjects		Out-patients		Healthy subjects		Out-patients	
	P-B-	P+B-^a^	PTB^b^	EPTB^c^	P-B-	P+B-^a^	PTB^b^	EPTB^c^
Treatment time (day)			30±7.38	32±8.22			31±6.52	35±7.46
Male (female) sex	16 (31)	20(27)	26(21)	24(23)	7(19)	11(22)	33(22)	50(25)
Age (yr)	28.72±9.45	39.83±5.69	44.23±12.78	35.22±14.62	28.92±8.84	40.06±5.74	39.49±11.05	35.20±10.50
Total number	47	47	47	47	26	33	55	75

Values were mean ± SD. a, b, c: there were 20, 25 and 34 subjects were used for both the serodiagnostic tests and the Interferon-γ release assays, respectively. P-B-, PPD (-) subjects without BCG vaccination; P+B-, PPD (+) subjects without BCG vaccination; PTB, Patients with pulmonary tuberculosis; EPTB, Patients with extrapulmonary tuberculosis

*Cell culture*: The cellular immune role of nHBHA was determined by IFN-γ secreted from PBMCs. Whole blood was collected from patients and volunteers after the TST was done. PBMCs from 4 ml human blood were prepared by density gradient separation on Ficoll-Paque Plus (Amersham Biosciences, USA). The isolated PBMCs were suspended at 2×10^6^ cells per ml in RPMI 1640 medium (GIBICO, USA) supplemented with 40 µg/ml of gentamin, 50 µM β-mercaptoethanol, 1×nonessential amino acid, 1×sodium pyruvate, 2 mM glutamine and 10 per cent foetal calf serum. PBMCs were cultured with HBHA (2 µg/ml), phytohaemaglutinin (PHA 4 µg/ml, positive control) and PBS (negative control) for four days^8^. The supernatants of the cultures were collected and stored until the IFN-γ assay was performed.

*Detection of the HBHA-specified antibody and IFN-γ by ELISA*: The optimal concentration of the rHBHA and nHBHA for coating the 96-well microplate was determined by the dilution method with murine antiserum. After the optimal concentration of HBHA protein was determined, the serum from each patient of PTB or EPTB and healthy P+B- and P-B- subjects was tested by enzyme-linked immunosorbent assay (ELISA)^8^. The concentration of IFN-γ in the culture supernatants was also measured by the ELISA method^7^ with an IFN-γ-detection kit (BD Biosciences, CA, USA) after the PBMCs were stimulated only by the nHBHA.

*Statistical analysis*: One-way analysis of variance (One-way ANOVA) by the software SPSS11.5 were used for the IFN-γ analysis after log transformation and to test for significance of the antibody IgG.

## Results

The ELISA results showed that the P+B- and P-B- subjects had low anti-HBHA. As expected, the healthy individuals (P+B- and P-B-) produced less anti-HBHA than TB patients (PTB and EPTB) when either rHBHA or nHBHA used in serodiagnostic tests. Between rHBHA and nHBHA, a better diagnosis on TB patients was possible with nHBHA as it produced higher titre in TB patients as compared to healthy individuals. nHBHA was used for detecting the HBHA specific antibody of PTB and EPTB subjects with a sensitivity of 90.26 and 91.67 per cent and specificity of 93.18 and 98.96 per cent, respectively (Table II).

**Table II.** Detection of the HBHA-specific antibody and the HBHA-specific IFN-γ production with nHBHA

		antibody level (OD)				log transformed IFN-γ level (pg/ml)			
		P-B- (n=47)	P+B- (n=47)	PTB (n=47)	EPTB (n=47)	P-B- (n=26)	P+B- (n=33)	PTB (n=55)	EPTB (n=75)
	Mean ± SD	237.80 ± 30.69	224.78 ± 40.82	317.94 ± 49.55	375.32 ± 60.36	1.47 ± 0.94	2.84 ± 0.93	2.25 ± 1.06	2.31 ± 0.96
P+B-	Cutoff value	0.2055				2.2218			
	Sensitivity (%) 95%CI	87.88 71.80 to 96.60				84.82 68.54 to 93.02			
	Specificity(%) 95%CI	76.92 56.35 to 91.03				80.71 65.22 to 92.62			
PTB	Cutoff value	0.2501	0.2490			1.8243	2.6346		
	Sensitivity (%) 95%CI	90.26 83.23 to 95.08	91.67 88.23 to 96.16			62.01 50.73 to 73.57	73.26 60.73 to 80.57		
	Specificity(%) 95%CI	93.18 86.76 to 97.14	93.75 89.36 to 98.34			60.57 42.14 to 77.09	71.84 51.14 to 82.09		
EPTB	Cutoff value	0.2805	0.2805	0.3555		1.8743	2.6843	2.1128	
	Sensitivity (%) 95%CI	91.67 88.72 to 99.14	91.67 88.72 to 99.14	77.08 62.69 to 87.97		64.01 54.24 to 78.51	71.01 59.54 to 80.83	53.33 43.26 to 74.38%	
	Specificity (%) 95%CI	98.96 89.23 to 99.04	100 91.78 to 100	87.50 74.75 to 95.27		61.45 48.63 to 78.28	68.45 50.63 to 85.28	58.37 48.22 to 75.65%	

OD, optical density measured at 450 nm; P-B-, PPD (-) subjects without BCG vaccination; P+B-, PPD (+) subjects without BCG vaccination; PTB, Patients with pulmonary tuberculosis; EPTB, Patients with extrapulmonary tuberculosis

The difference in anti-HBHA levels in patients with PTB and EPTB was significant (*P*<0.05 for rHBHA and for nHBHA), but both proteins (rHBHA and nHBHA) detected PTB and EPTB with the limited sensitivity of 77.08 per cent (for both rHBHA and nHBHA, 95%CI, 62.69 to 87.97%) and specificity of 81.25 per cent (rHBHA, 95%CI, 67.37 to 91.05%) and 87.5 per cent (nHBHA, 95%CI, 74.75 to 95.27%). Overall, a cut-off values of OD _450_0.3485 and 0.3575 were employed for rHBHA and nHBHA analyses, respectively.

The results of the serodiagnostic tests showed that the patients with PTB and EPTB could develop the humoral response against HBHA. The nHBHA protein could partly differentiated PTB and EPTB, however, failed between P+B- and P-B- subjects. Instead, IGRA showed the potential for differentiating the P+B- subjects with the P-B- subjects (Table II). IFN-γ levels between the healthy P+B- and P-B- groups were significantly different (*P*< 0.001) with a detection sensitivity of 84.82 per cent (95%CI, 68.54 to 93.02%) and specificity of 80.71 per cent (95%CI, 65.22 to 92.62%). PBMCs from the P+B- subjects produced higher HBHA-induced IFN-γ than PTB patients and EPTB patients, but they were not significantly different. The PTB and EPTB subjects showed no difference in IFN-γ production. Both were differentiated with poor sensitivity of 53.33 per cent (95%CI, 43.26 to 74.38%) and specificity of 58.37 per cent (95%CI, 48.22 to 75.65%).

## Discussion

The mycobacterium HBHA is a surface-expressed adhesion that can affect binding to host epithelial cells via a unique Lys-Pro-Ala-rich C-terminal region^1^,^7^,^15^. Post-translational methylated modification in this region is very critical for generating effective host immune responses^5^,^7^. Patients with TB usually develop a strong humoral response against nHBHA^7^, but little is known about the difference of anti-HBHA responses between patients with PTB and EPTB. Here, the usefulness and effectiveness of humoral anti-HMHA immune responses was studied in the clinical diagnosis of tuberculosis. The results showed that HBHA in serodiagnostic tests has the limited potential ability to discriminate healthy individuals with latent TB infection from TB patients, and patients with PTB from EPTB with a limited sensitivity and specificity.

Some reports suggested that nHBHA could induce significantly higher IFN-γ production by PBMC from P+B- subjects as compared to P-B- controls and TB patients^5^,^9^. Our results on the nHBHA specific IGRAs also showed an increase in IFN-γ production in patients (PTB and EPTB), compared to the P-B- healthy individuals, but the specificity and sensitivity were low. Our results of nHBHA-induced IFN-γ production did not discriminate the P+B- subjects from the patients with PTB and EPTB after log transformation. This finding was different from an earlier report that lymphocytes from 60 per cent of healthy infected individuals produced IFN-γ after stimulation with HBHA, compared with only 4 per cent of patients with active TB^9^.

EPTB is difficult to diagnose since the sputum smears of these patients are negative. X-rays also cannot offer the aetiology of the disease; therefore nHBHA has been suggested for diagnosing EPTB^3^,^7^. Our results showed its limited power in the clinical diagnosis and also showed certain disadvantages such as the laborious isolation of PBMCs and cell culture, the low positive rate of either serodiagnostic test or IGRAs, and the uncertainty of BCG vaccination and environmental mycobacterial exposure history. Further investigations on the accurate microbiological and clinical relationships and the use of the HBHA on different types of EPTB are required.

## References

[1] (2009). World Health Organization. *Global tuberculosis control: Epidemiology, strategy, financing. WHO/HTM/TB/2009.411*.

[2] Menozzi FD, Rouse JH, Alavi M, Laude-Sharp M, Muller J, Bischoff R (1996). Identification of a heparin-binding hemagglutinin present in mycobacteria. *J Exp Med*.

[3] Pethe K, Alonso S, Biet F, Delogu G, Brennan MJ, Locht C (2001). The heparin-binding hemagglutinin of *M.tuberculosis* is required for extrapulmonary dissemination. *Nature*.

[4] Pethe K, Bifani P, Drobecq H, Sergheraet C, Debrie A, Locht C (2002). Mycobacterial heparin-binding hemagglutinin and laminin-binding protein share antigenic methyllysines that confer resistance to proteolysis. *Proc Natl Acad Sci USA*.

[5] Temmerman S, Pethe K, Parra M, Alonso S, Rouanet C, Pickett T (2004). Methylation-dependent T cell immunity to *Mycobacterium tuberculosis* heparin-binding hemagglutinin. *Nat Med*.

[6] Reddy VW, Kumar B (2000). Interaction of *Mycobacterium avium* complex with human respiratory epithelial cells. *J Infect Dis*.

[7] Parra M, Pickett T, Delogu G, Dheenadhayalan V, Debrie A, Locht C (2004). The Mycobacterial heparin-binding hemagglutinin is a protective antigen in the mouse aerosol challenge model of tuberculosis. *Infect Immun*.

[8] Zanetti S, Bua A, Delogu G, Pusceddu C, Mura M, Saba F (2005). Patients with pulmonary tuberculosis develop a strong humoral response against methylated heparin-binding hemagglutinin. *Clin Diagn Lab Immun*.

[9] Masungi C, Temmerman S, Van Vooren JP, Drowart A, Pethe K, Menozzi FD (2002). Differential T and B cell responses against *Mycobacterium tuberculosis* heparin-binding hemagglutinin adhesion in infected healthy individuals and patients with tuberculosis. *J Infect Dis*.

[10] Menzies D, Pai M, Comstock G (2007). Meta-analysis: new tests for the diagnosis of latent tuberculosis infection: areas of uncertainty and recommendations for research. *Ann Intern Med*.

[11] Richeldi L (2006). An update on the diagnosis of tuberculosis infection.*Am*. *J Respir Crit Care Med*.

[12] Shin AR, Lee KS, Lee JS, Kim SY, Song CH, Jung SB (2006). *Mycobacterium tuberculosis* HBHA protein reacts strongly with the serum immunoglobulin M of tuberculosis patients. *Clin Vaccine Immunol*.

[13] Sambrook J, Russell DW (2001). *Molecular cloning: A laboratory manual*.

[14] Baulard A, Kremer L, Locht C (1996). Efficient homologous recombination in fast-growing and slow-growing mycobacteria. *J Bacteriol*.

[15] Menozzi FD, Bischoff RR, Fort E, Brennan MJ, Locht C (1998). Molecular characterization of the Mycobacterial heparin-binding hemagglutinin, a mycobacterial adhesion. *Proc Natl Acad Sci USA*.

[16] Chattopadhayay R, Kaur S, Ganguly NK, Mahajan RC (1996). Antigenic differences between axenic amastigotes & promastigotes of Leishmania donovani. *Indian J Med Res*.

